# Correlates of Unintended Pregnancy in Ethiopia: Results From a National Survey

**DOI:** 10.1371/journal.pone.0082987

**Published:** 2013-12-09

**Authors:** Dereje Habte, Sisay Teklu, Tadele Melese, Mgaywa G. M. D. Magafu

**Affiliations:** 1 School of Public Health, University of Botswana, Gaborone, Botswana; 2 Department of Gynecology/ Obstetrics, Addis Ababa University, Addis Ababa, Ethiopia; 3 School of Medicine, University of Botswana, Gaborone, Botswana; Iran University of Medical Sciences, Islamic Republic of Iran

## Abstract

**Background:**

Unintended pregnancy has been a major reproductive health challenge in resource poor settings including Ethiopia. It has adverse consequences to the mother, child and the health sector’s resources. Understanding the extent of unintended pregnancy and the factors associated is crucial to devise evidence based interventions. The analysis was aimed at assessing the unintended pregnancy prevalence rate among pregnant women and the factors predisposing to unintended pregnancy.

**Methods:**

This secondary data analysis was done on women’s dataset from the 2011 Ethiopian Demographic and Health Survey (DHS). A total of 1267 pregnant women were included in the analysis. Multiple logistic regression analysis was performed using SPSS software to identify the factors associated with unintended pregnancy. Odds Ratio with 95% confidence interval (95% CI) was computed to assess the association of different factors with unintended pregnancy.

**Results:**

The overall prevalence of unintended pregnancy was found to be 24%: those who wanted it at a later time and not at all accounted for 17.1% and 6.9%, respectively. The unintended pregnancy rate ranged from 1.5% in Afar Regional State to 39.8% in Oromiya Regional State. Women who knew the timing of ovulation had a 45% reduced chance of unintended pregnancy (OR (95% CI): 0.55 (0.35, 0.85)). Ever use of family planning, presence of five or more born children, and two or more births in the past five years were associated with unintended pregnancy (OR (95% CI): 1.79 (1.31, 2.45), 2.36 (1.01, 5.49) and 2.00 (1.12, 3.58), respectively).

**Conclusions:**

A significant proportion of the current pregnancies were found to be unintended with significant variations among the different regions. Women already burdened with higher fertility were suffering from unintended pregnancy. Family planning programs need to concentrate on the highly affected regions and target women with higher fertility to reduce the level of unintended pregnancy at national level.

## Introduction

The concept of unintended pregnancy has been employed by demographers and public health practitioners in addressing women’s health. Unintended pregnancy refers to pregnancies that are reported to have been either unwanted (not wanted at the time) or mistimed (wanted but at a later time) [1]. Globally it is estimated that there are 87 million cases of unintended pregnancies annually of which 46 million cases resort to induced abortion. Not all cases have access to safe abortion facilities and 18 million cases end up with unsafe abortion services [2]. With the increased use of contraceptives over the years, there has been a decreasing trend in the global unintended pregnancy rate which declined from 69 per 1,000 women 15-44 years age in 1995 to 55 per 1000 women 15-44 years age in 2008. The decline in Africa was far less than the reduction in the developed world: 92 per 1,000 women 15-44 years age in 1995 to 86 per 1000 women 15-44 years age in 2008 [3]. 

Studies have shown that there are a number of factors predicting the occurrence of unintended pregnancies. The socio-demographic factors that were reported to have been associated with increased level of unintended pregnancy included younger age, less level of education, unmarried, rural residence, and lower income. Distance from the nearest health facility, higher parity, previous history of unintended pregnancy, unmet need for family planning, family planning method failure, early sexual initiation, partner’s desire for child, domestic violence and less autonomy were among the predictors of unintended pregnancy [4-15]. 

Unintended pregnancy has been a major public health issue due to its adverse consequences to the mother, child and the health sector’s resources [2]. The pre-natal adverse effect includes medical complications and maternal mortality secondary to complicated abortion. For those pregnancies not terminated, they might encounter violence, delayed ante-natal care initiation and unsafe delivery service utilization. The born children were also shown to be affected in terms of child care, psycho-social development and health status [16-20].

In Ethiopia, a nationwide survey conducted at interval of 5-6 years revealed a decreasing trend in the percentage of unwanted pregnancies: 17 %, 16% and 9% in 2000, 2005 and 2011 respectively. However, the percentage of births that were wanted later remained stable over the years in the range of 19-20% [21]. Studies done in the different regions of Ethiopia revealed unintended pregnancy rate in the range between 27.9% and 42.4% [6-9]. 

This report is based on analysis of existing data from a nationwide demographic and health survey conducted in 2011 [21]. The study represents all the regional states including the urban centers. The analysis is aimed at assessing the prevalence of unintended pregnancy and the factors associated with unintended pregnancy among pregnant women in the Ethiopian Demographic and Health Survey (EDHS)-2011 dataset to avail evidence for future decision making.

## Materials and Methods

### Study area and data collection

Ethiopia is administratively divided into nine regional states and two city administrations that are further divided into 16,253 Kebeles, the smallest administrative units in the administrative structure of the country. The EDHS covers all the administrative regions (nine regional states and two city administrations) in the country. Data for this study was drawn from the third Ethiopian Demographic and Health Survey - 2011. The survey was implemented by the Central Statistics Authority, Ethiopia with the technical support of ORC Macro. The survey collected information from a nationally representative sample of 16702 households, 16515 women (15-49 years), 15908 men (15-59 years) and 11569 children under-five. The sample for the 2011 EDHS was designed to provide population and health indicators at the national (urban and rural) and regional levels [21].

The 2011 EDHS used questionnaires that were adapted from model survey instruments developed for the MEASURE DHS project. Various stakeholders were consulted to improve on the tool and the questionnaires were translated into three major languages—Amharigna, Oromiffa, and Tigrigna. The questionnaire was pre-tested before the start of the field work in all the three local languages to make sure that the questions were clear and understandable to the respondents. The questionnaires were modified following the input of the pre-test. Training was conducted for the interviewers, editors and supervisors for one month. The training consisted of instruction on interviewing techniques and field procedures, and a detailed review of the questionnaire content. Quality control teams regularly visited and often stayed with the teams throughout the fieldwork period to closely supervise and monitor the data collection [21].

In 2011 the Ethiopian DHS recorded data relating to: family planning; fertility levels and determinants; fertility preferences; infant, child, adult and maternal mortality; maternal and child health; nutrition; women’s empowerment and knowledge of HIV/AIDS. The analysis is based on 1267 women who were pregnant at the time of data collection. Retrospectively reported pregnancy intentions for past pregnancies generally become more positive as mothers tend to like the baby once born [22]. Hence this analysis was made only on women who were pregnant at the time of data collection. 

### Outcome measure

Women were asked if the current pregnancy was wanted or not. If the pregnancy was wanted then, it is considered to be planned. It is considered to be mistimed if it was wanted but at a later time; and unwanted if it was not wanted at the time. Mistimed and unwanted pregnancies were merged as “unintended” to create a binary variable with the planned pregnancies (intended). 

### Exposure measures

The potential predictors of unintended pregnancy identified in the dataset were grouped in to socio-demographic, reproductive and autonomy related variables.

• Socio-demographic characteristics of women: Age, Residence: Urban/ Rural, Education, Marital status, Wealth Index
• Reproductive Health: Ideal number of children, Number of children ever born, Number of living children, Entry birth order, Number of births in the past five years, Ever terminated pregnancy, Knowledge of ovulation timing, Ever use of family planning, Partner desire for children
• Autonomy is represented by selected direct measures of women’s autonomy [23]: decision-making power on woman’s visit to families or relatives, decision-making power on making large household purchases, decision-making power on woman’s health care; and women’s attitudes toward wife beating. The response categories for decision making variables are “respondent alone”, “jointly with partner” and “partner/someone else”.

### Analysis

Percentage and mean/ median were used to describe the socio-demographic and reproductive characteristics of study participants. Bivariate analysis in terms of Chi-square test has been used to assess the effect of each independent variable towards un-intended pregnancy. Ideal number of children, number of children ever born, number of living children and entry birth order demonstrated correlation. To avoid multi-collinearity, only “number of children ever born” was selected to be included in the logistic regression analysis. Independent variables with p-value less than 0.25 in the bivariate analysis were included in the multiple logistic regression analysis to control for possible confounding factors [24]. Multiple logistic regression analysis (Enter method) was used, and Odds Ratio with 95% Confidence Interval (95 % CI) computed to describe the association of risk factors with unintended pregnancy. Statistical significance was considered at p-value less than 0.05. SPSS software (version 20.0) was used in the analysis of the data.

### Ethical review

Ethical clearance for the survey was provided by the Ethiopian Health and Nutrition Research Institute Review Board, the National Research Ethics Review Committee at the Ministry of Science and Technology, the Institutional Review Board of ICF International, and the Center for Disease Control [21].

Detailed information on the study area, study population, organization of the survey, sample design, questionnaires, data collection, data quality, data processing and ethical issue is published in the Ethiopian Demographic and Health Survey 2011 report [21].

The primary author communicated with MEASURE DHS/ ICF International and permission was granted to download and use the data for this project.

## Result

### Socio-demographic characteristics

Table 1 summarizes the socio-demographic characteristics of study participants. A total of 1267 currently pregnant women in the 2011 Ethiopian Demographic and Health Survey were included in the analysis 3-fourth of the respondents were between 20 and 34 years of age with overall mean age of 27.5. The large majorities of the women were rural residents and married. Two-third never had formal education while half of them belonged to the poor wealth index categories. 

**Table 1:** Socio-demographic characteristics of pregnant study participants, Ethiopia, 2011.

**Characteristics**		**Frequency**	**Percentage**
**Age in years**	15-19	129	10.2
	20-24	289	22.8
	25-29	373	29.4
	30-34	253	20.0
	35-39	154	12.2
	40-44	57	4.5
	45-49	12	0.9
	Mean (SD): 27.5 (6.5)		
**Type of place of residence**	Urban	244	19.3
	Rural	1023	80.7
**Marital status**	Married	1154	91.1
	Living with partner	82	6.5
	Widowed	4	0.3
	Divorced	10	0.8
	Separated	10	0.8
	Never in union	7	0.6
**Highest education level**	No formal education	804	63.5
	Primary	384	30.3
	Secondary	45	3.6
	Tertiary	34	2.7
**Wealth index**	Poorest	399	31.5
	Poorer	208	16.4
	Middle	182	14.4
	Richer	205	16.2
	Richest	273	21.5

### Reproductive characteristics

**Figure 1:**
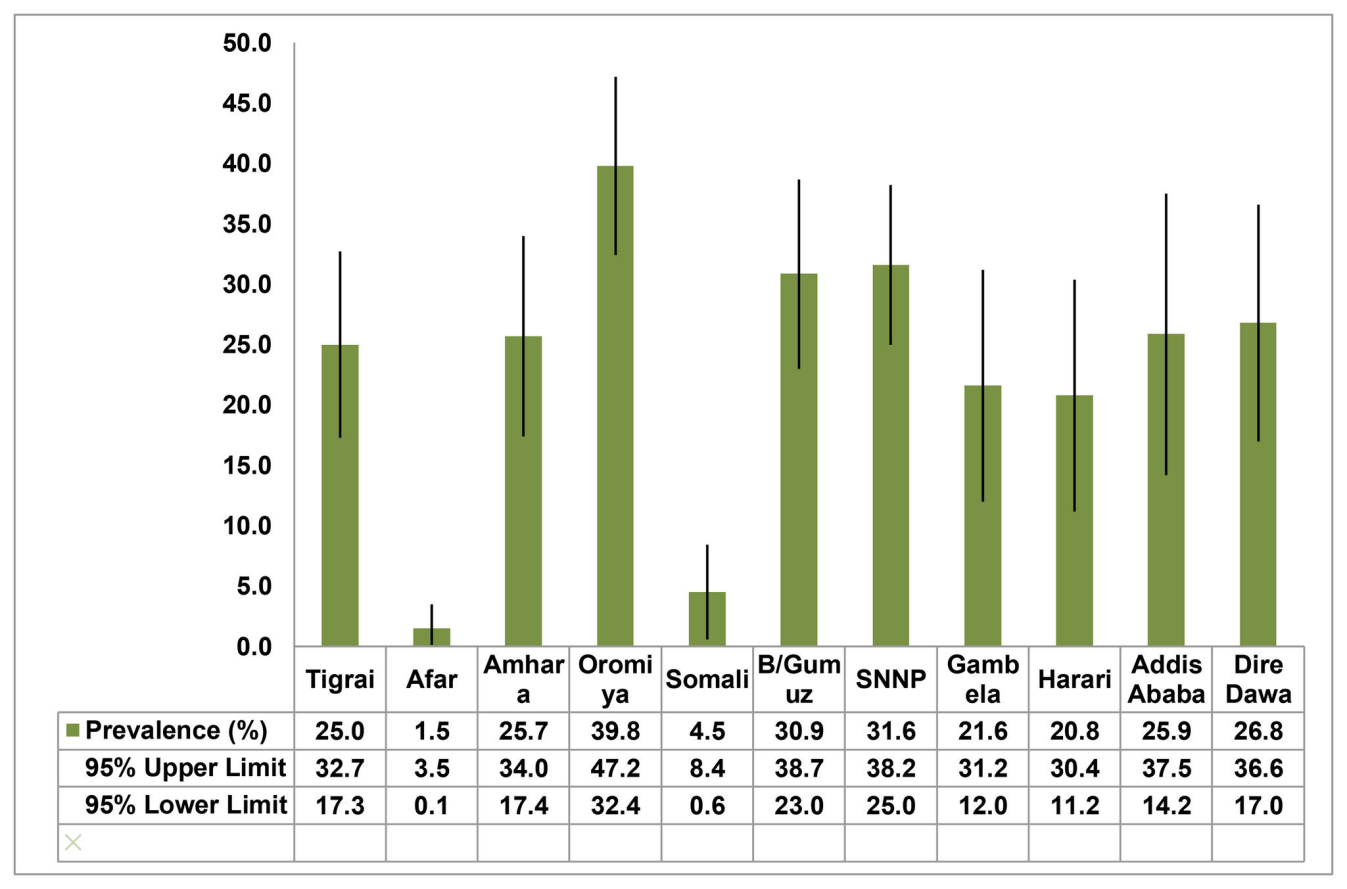
The prevalence of unintended pregnancy in the 11 Regions of Ethiopia, 2011.

Over three-fourth of the respondents reported that the current pregnancy is wanted at that point while those who wanted it at a later time and not at all accounted for 17.1% and 6.9%, respectively. The unintended pregnancy rate ranged from 1.5% in Afar Regional State to 39.8% in Oromiya Regional State. The number of children ever born ranged from 0 to 12 with a median of three. One-fourth of the participants had no birth in the five years preceding the survey. One-third of the women reported their desired number of children to be 6 or more whereas 14.1% gave a non-numeric response (“God/ Alah knows” or “Don’t know”). Respondents who knew of modern contraceptives constituted 93.3% while 15% recognized that ovulation time is at the middle of the cycle. Ever use of any family planning method was reported by 30.1% of the respondents. Over one-third of the women replied that both partners desire the same number of children where as one-quarter stated that the partner wants more number of children. (Figure 1 and Table 2)

**Table 2:** Reproductive characteristics of pregnant study participants, Ethiopia, 2011.

**Characteristics**		**Frequency**	**Percentage**
**Current pregnancy wanted**	Planned	962	75.9
	Mistimed	217	17.1
	Unwanted	88	6.9
**Number of children ever born**	0	213	16.8
	1-2	405	32.0
	3-4	303	23.9
	5+	346	27.3
	Median (IQR): 3 (4)		
**Number of births in past five years**	0	340	26.8
	1	619	48.9
	2	261	20.6
	3	44	3.5
	4	3	0.2
	Median (IQR): 1 (1)		
**Ideal number of children**	0	72	5.7
	1	8	0.6
	2	74	5.8
	3	71	5.6
	4	295	23.3
	5	126	9.9
	6+	442	34.9
	Non-numeric response	179	14.1
**Knowledge of family planning method**	Knows no method	81	6.4
	Knows only traditional method	4	0.3
	Knows modern method	1182	93.3
**Ever used any family planning method**	Yes	389	30.7
	No	878	69.3
**Knowledge on timing of ovulation**	During her period	28	2.2
	After period ended	382	30.1
	Middle of the cycle	190	15.0
	Before period begins	51	4.0
	At any time	299	23.6
	Other	2	0.2
	Don't know	315	24.9
**Partners’ Desire for children**	Both want same	485	38.3
	Husband wants more	317	25.0
	Husband wants fewer	115	9.1
	Don't know	319	25.2
	No response	31	2.4

### Bi-variate analysis of exposure measures

Table 3 and Table 4 describe the bi-variate analysis result between the exposure measures (socio-demographic, reproductive and decision making) and the outcome measure (unintended pregnancy). Level of education and wealth index were among the socio-demographic variables that showed significant association with unintended pregnancy (p<0.05). Among the reproductive characteristics, the variables that demonstrated significant relationship with unintended pregnancy included knowledge on the timing of ovulation, ever use of family planning, number of children ever born and number of births in the five years preceding the survey (p<0.05). Partner’s desire for children, decision making power and wife beating variables did not show any statistically significant association in the bi-variate analysis (p>0.05).

**Table 3:** Bivariate analysis of socio-demographic and reproductive characteristics versus unintended pregnancy, Ethiopia, 2011.

**Characteristics**		**Unintended pregnancy Number (Row %)**		**Chi-Square (p-value)**
		**Yes**	**No**	
**Age in years**				
	15-19	35 (27.1)	94 (72.9)	6.96 (0.13)
	20-24	66 (22.8)	223 (77.2)	
	25-29	83 (22.3)	290 (77.7)	
	30-34	54 (21.3)	199 (78.7)	
	35-49	67 (30.0)	156 (70.0)	
**Type of place of residence**				
	Urban	50 (20.5)	194 (79.5)	2.12 (0.14)
	Rural	255 (24.9)	768 (75.1)	
**Highest education level**				
	No formal education	164 (20.4)	640 (79.6)	28.39 (0.00)
	Primary	129 (33.6)	255 (66.4)	
	Secondary and above	12 (15.2)	67 (84.8)	
**Wealth index**				
	Poorest	69 (17.3)	330 (82.7)	22.24 (0.00)
	Poorer	58 (27.9)	150 (72.1)	
	Middle	54 (29.7)	128 (70.3)	
	Richer	65 (31.7)	140 (68.3)	
	Richest	59 (21.6)	214 (78.4)	
**Ever terminated pregnancy**				
	Yes	41 (25.0)	123 (75.0)	0.08 (0.76)
	No	264 (23.8)	839 (76.2)	
**Knows the timing of ovulation**				
	Yes	32 (16.8)	158 (83.2)	6.39 (0.01)
	No	273 (25.3)	804 (74.7)	
**Ever use of family planning**				
	Yes	116 (29.8)	273 (70.2)	10.14 (0.00)
	No	189 (21.5)	689 (78.5)	
**No of children ever born**				
	0	40 (18.8)	173 (81.2)	11.76 (0.00)
	1-2	89 (22.0)	316 (78.0)	
	3-4	71 (23.4)	232 (76.6)	
	5+	105 (30.3)	241 (69.7)	
**Number of births in past five years**				
	0	64 (18.8)	276 (81.2)	7.65 (0.02)
	1	156 (25.2)	463 (74.8)	
	2-4	85 (27.6)	223 (72.4)	

**Table 4:** Bivariate analysis of partner’s fertility desire and decision making variables versus unintended pregnancy, Ethiopia, 2011.

**Characteristics**		**Unintended pregnancy Number (Row %)**		**Chi-Square (p-value)**
		**Yes**	**No**	
**Partners’ Desire for children**				
	Both want same	118 (24.3)	367 (75.7)	1.11 (0.77)
	Husband wants more	76 (24.0)	241 (76.0)	
	Husband wants fewer	28 (24.3)	87 (75.7)	
	Don't know	68 (21.3)	251 (78.7)	
**Decision on visiting families/relative**				
	Respondent alone	54 (22.4)	187 (77.6)	0.50 (0.77)
	Jointly with partner	160 (24.7)	488 (75.3)	
	Partner/someone else	91 (24.1)	287 (75.9)	
**Decision on large household purchases**				
	Respondent alone	19 (24.7)	58 (75.3)	0.29 (0.86)
	Jointly with partner	161 (23.5)	525 (76.5)	
	Partner/someone else	125 (24.8)	379 (75.2)	
**Decision on woman’s health care**				
	Respondent alone	49 (27.5)	129 (72.5)	1.81 (0.40)
	Jointly with partner	154 (22.8)	521 (77.2)	
	Partner/someone else	102 (24.6)	312 (75.4)	
**Wife beating justifiable**				
	Yes	213 (23.3)	700 (76.7)	0.98 (0.32)
	No	92 (26.0)	262 (74.0)	

### Multiple logistic regression analysis of exposure measures

Table 5 presents the result of the multiple logistic regression analysis for the socio-demographic and fertility variables with p-value less than 0.25 in the bi-variate analysis. Women in the age groups 25-29 years and 30-34 years had a reduced chance of unintended pregnancy as compared to the 15-19 years age group (OR (95% CI): 0.46 (0.25, 0.83) and 0.40 (0.20, 0.78), respectively). Women in primary education category were more likely to have unintended pregnancy as compared to the no education category (OR (95% CI): 2.38 (1.73, 3.26)). Women who knew the timing of ovulation had a 45% reduced chance of unintended pregnancy (OR (95% CI): 0.55 (0.35, 0.85)). Ever use of family planning, presence of five or more born children, and two or more deliveries in the past five years were associated with unintended pregnancy (OR (95% CI): 1.79 (1.31, 2.45), 2.36 (1.01, 5.49) and 2.00 (1.12, 3.58), respectively). 

**Table 5:** Factors predicting unintended pregnancy among pregnant women (Logistic regression analysis), Ethiopia, 2011.

**Variables**		Adjusted Odds Ratio* (95% CI)	**p-value**
**Age in years**	15-19	**Reference**	
	20-24	0.63 (0.37, 1.09)	0.10
	25-29	0.46 (0.25, 0.83)	0.01
	30-34	0.40 (0.20, 0.78)	0.00
	35-49	0.59 (0.28, 1.22)	0.15
**Education**	No formal education	**Reference**	
	Primary	2.38 (1.73, 3.26)	0.00
	Secondary & above	1.49 (0.70, 3.19)	0.29
**Knows the timing of ovulation**	Yes	0.55 (0.35, 0.85)	0.00
	No	**Reference**	
**Ever use of family planning**	Yes	1.79 (1.31, 2.45)	0.00
	No	Reference	
**No of children ever born**	0	**Reference**	
	1-2	1.02 (0.51, 2.01)	0.94
	3-4	1.45 (0.66, 3.21)	0.35
	5+	2.36(1.01, 5.49)	0.04
**No of deliveries in 5 years**	0	**Reference**	
	1	1.65 (0.98, 2.78)	0.05
	2-4	2.00 (1.12, 3.58)	0.01

^*^ Adjusted for place of residence and wealth index.

## Discussion

The data analyzed included all the regions in Ethiopia with urban and rural representation. In the current analysis, 24 % of the pregnancies were unintended. The analysis was focused on the current pregnancy with the intention of minimizing both recall bias and underestimation of unintended pregnancy if one uses previous pregnancies [22]. The current rate is less than the reports from the small scale studies done in the different parts of Ethiopia that ranged between 27.9% and 42.4% [6-9]. The analysis revealed that some regional states have extremely low level of un-intended pregnancy that resulted in the lowering of national average. The significant difference in the extent of unintended pregnancy between regions calls for the need of targeted interventions based on the enormity of the problem. 

The Ethiopian Census 2007 report estimated that there were over 17.2 million females in the age range 15-49 years residing in Ethiopia [25]. A research in Ethiopia revealed a pregnancy rate of 227/year/1000 women of reproductive age [26]. Nearly four million pregnant mothers are expected in Ethiopia over one year duration. Considering the 24% unintended current pregnancy level at national level, nearly one million unintended pregnancies are expected to occur each year in Ethiopia. Reproductive health programs need to play a key role to significantly reduce such a huge number of unintended pregnancies in Ethiopia. It is one way of addressing the reproductive right of individuals and couples to access family planning services and hence minimize unintended pregnancies. If the women had had access to fertility control services, such huge number of unintended pregnancies wouldn’t have occurred. The nation seems not on progress in terms of fulfilling one of the reproductive health rights namely access to safe, effective, affordable, and acceptable methods of family planning.

Women in the age groups 25 to 34 years were found to have a significantly less level of unintended pregnancy as compared to teen agers which might be explained by the possible increased level of reproductive health knowledge and access to family planning services. Women in that age range are also more likely to be in a marital union and hence the pregnancies are more likely to be intended. It can also be ascribed to more desire for children at a prime age of fertility and hence the pregnancy is more likely to be planned. The age groups 20 to 24 years and 35 to 49 years also had a reduced level of unintended pregnancy although not statistically significant. Primary education level was shown to be associated with increased level of unintended pregnancy as compared to those women with no formal education. Better education was thought to reduce the chance of having unintended pregnancy which was not the case in the current analysis. Educational background has also been shown to have either insignificant or inconsistent relationship with unintended pregnancy in diverse settings [6-12].

The knowledge of family planning method was 93.6% where as ever use of any family planning method being 30.1%. There is a huge discrepancy and knowledge does not seem to guarantee the practice of family planning. In addition, women who ever used any family planning were significantly more likely to report unintended pregnancy in conformity with study findings in Bangladesh and Nigeria [4,12]. Women who ever used family planning might have utilized it long time ago and hence couldn’t predict recent pregnancy outcome. It appears advisable to ask for a detailed information on ever use of family planning in terms of the timing. If the “ever use” is at a recent time, one can also think of contraception failure or discontinuation. The current dataset does not specify about the timing of past family planning use that would have helped to differentiate between recent and older exposure to family planning. 

Knowledge on the timing of ovulation demonstrated a significant reduction in unintended pregnancy. Such association might be difficult to interpret but it would be advisable to investigate if such groups of women had been using their knowledge of ovulation timing for fertility control (Natural Method). Women with five or more children ever born demonstrated increased chance of unintended pregnancy in conformity with other studies done in Ethiopia and elsewhere [6-9,11,12]. Women with history of two or more pregnancies in the five years preceding the survey were also more likely to have unintended pregnancy. It is an indication that there are women who continued living at risk of pregnancy despite attaining high level of fertility at one point in time. Different factors might have contributed for the failure of such women in avoiding unintended pregnancy. Such women with higher fertility are more likely to have unmet need for family planning. The factors predisposing the women to the risk of pregnancy might be related to the woman, partner, family, society, health worker or health program performance. In-depth investigation on such women is advised to assess the root cause for their failure that can help in developing evidence based intervention. Health programs have to devise a mechanism of reaching women with increased level of fertility who desire to space or limit birth.

Partner’s desire for children, decision making power and wife beating variables were not found to be associated with unintended pregnancy. Different studies revealed that partner’s influence had a significant association with unintended pregnancy [8,9,15]. Although there is no significant finding at national level, one can’t rule out regional differences as autonomy related characteristics differ between regions in Ethiopia. Further analysis to check for any regional difference regarding partners’ influence on fertility was not possible due to the relatively small number of participants per region.

The limitation of this study is that relevant variables such as cultural influence, accessibility to health service, communication between spouses, and reasons for the failure to avoid unintended pregnancy were not available in the dataset for analysis. Nevertheless the strength of the DHS data lies on the representation of diverse population groups, wide geographic coverage, use of standard questionnaire and data processing. 

## Conclusion

A significant proportion of the current pregnancies were found to be unintended. Women already burdened with higher fertility were suffering from unintended pregnancy. Regional differences of unintended pregnancy were noted. Family planning programs need to target women with higher fertility to minimize the risk of unintended pregnancy. Reproductive Health Managers are advised to investigate the effectiveness of the existing family planning program in reaching such groups of women. Targeted interventions need to be implemented in the regions with higher level of unintended pregnancy. Further in-depth investigation is recommended to identify the major gaps to be addressed in the prevention of unintended pregnancy.

## References

[1] Santelli J, Rochat R, Hatfield-Timajchy K, Gilbert BC, Curtis K et al. (2003) The measurement and meaning of unintended pregnancy. Perspect Sex Reprod Health 35: 94-101. doi:10.1363/3509403. PubMed: 12729139. 12729139

[2] World Health Organization (2009) World health report 2005: make every mother and child count. WHO, Geneva, 2005

[3] Singh S, Sedgh G, Hussain R (2010) Unintended pregnancy: worldwide levels, trends, and outcomes. Stud Fam Plann 41: 241-250. doi:10.1111/j.1728-4465.2010.00250.x. PubMed: 21465725. 21465725

[4] Kamal M, Islam A (2011) Prevalence and socioeconomic correlates of unintended pregnancy among women in rural Bangladesh. Salud Publica Mexico 53: 108-115. doi:10.1590/S0036-36342011000200003. 21537801

[5] Magadi MA (2003) Unplanned childbearing in Kenya: the socio-demographic correlates and the extent of repeatability among women. Soc Sci Med 56(1): 167-178. PubMed: 12435559. 12435559 10.1016/s0277-9536(02)00018-7

[6] Worku S, Fantahun M (2007) Unintended pregnancy and induced abortion in a town with accessible family planning services: The case of Harar in eastern Ethiopia. Ethiopian Journal of Health Development 20(2): 79-83.

[7] Kassa N, Berhane Y, Worku A (2010) Predictors of unintended pregnancy in Kersa, Eastern Ethiopia. Reproductive Health 9(1): 1-7. 10.1186/1742-4755-9-1 PMC3320539 22239744

[8] Hamdela B, Tilahun T (2012) Unwanted Pregnancy and Associated Factors among Pregnant Married Women in Hosanna Town, Southern Ethiopia. PLOS ONE 7(6): e39074. doi:10.1371/journal.pone.0039074.

[9] Geda NR, Lako TK (2011) A population based study on unintended pregnancy among married women in a district in Southern Ethiopia. Journal Geography and Regional Planning 4(7): 417-427.

[10] Goto A, Yasumura S, Reich MR, Fukao A (2002) Factors associated with unintended pregnancy in Yamagata, Japan. Soc Sci Med 54(7): 1065-1079. PubMed: 11999503. 11999503 10.1016/s0277-9536(01)00081-8

[11] Ikamari LD, Izugbara C, Ochako R (2013) Prevalence and determinants of unintended pregnancy among women in Nairobi, Kenya. BMC Pregnancy Childbirth 13(1): 69. doi:10.1186/1471-2393-13-69. PubMed: 23510090. 23510090 PMC3607892

[12] Sedgh G, Bankole A, Oye-Adeniran B, Adewole IF, Singh S et al. (2006) Unwanted pregnancy and associated factors among Nigerian women. Int Fam Plan Perspect 32(4): 175-184. doi:10.1363/3217506. PubMed: 17237014. 17237014

[13] Henshaw SK (1998) Unintended pregnancy in the United States. Fam Plann Perspect. 30(1): 24-46. doi:10.2307/2991522. PubMed: 9494812. 9494812

[14] Tsui AO, McDonald-Mosley R, Burke AE (2010) Family planning and the burden of unintended pregnancies. Epidemiol Rev 32(1): 152-174. doi:10.1093/epirev/mxq012. PubMed: 20570955. 20570955 PMC3115338

[15] Kaye DK, Mirembe FM, Bantebya G, Johansson A, Ekstrom AM (2006) Domestic violence as risk factor for unwanted pregnancy and induced abortion in Mulago Hospital, Kampala, Uganda. Trop Med Int Health 11(1): 90-101. PubMed: 16398760. 16398760 10.1111/j.1365-3156.2005.01531.x

[16] Grimes DA, Benson J, Singh S, Romero M, Ganatra B, et al (2006) Unsafe abortion: The preventable pandemic. Lancet 368(9,550): 1,908–1,919 10.1016/S0140-6736(06)69481-6 17126724

[17] Marston C, Cleland J (2003) Do unintended pregnancies carried to term lead to adverse outcomes for mother and child? An assessment in five developing countries. Popul Stud (Camb) 57(1): 77-93. doi:10.1080/0032472032000061749. PubMed: 12745811. 12745811

[18] Exavery A, Kanté AM, Hingora A, Mbaruku G, Pemba S et al. (2013) How mistimed and unwanted pregnancies affect timing of antenatal care initiation in three districts in Tanzania. BMC Pregnancy Childbirth 13(1): 35. doi:10.1186/1471-2393-13-35. PubMed: 23388110. 23388110 PMC3574825

[19] Kubicka L, Matĕjcek Z, David HP, Dytrych Z, Miller WB et al. (1995) Children from unwanted pregnancies in Prague, Czech Republic revisited at age thirty. Acta Psychiatr Scand 91: 361–369. doi:10.1111/j.1600-0447.1995.tb09795.x. PubMed: 7676833. 7676833

[20] Myhrman A (1988) Family relation and social competence of children unwanted at birth. Acta Psychiatr Scand 77: 181–187. doi:10.1111/j.1600-0447.1988.tb05098.x. PubMed: 3364204. 3364204

[21] Central Statistical Agency (2011) Ethiopia Demographic and Health Survey. Central Statistical Agency, Addis Ababa, Ethiopia ORC Macro Calverton, Maryland, USA

[22] Joyce TJ, Kaestner R, Korenman S (2000) The stability of pregnancy intentions and pregnancy-related maternal behaviors. Matern Child Health J 4(3): 171–178. doi:10.1023/A:1009571313297. PubMed: 11097504. 11097504

[23] Woldemicael G (2010) Do women with higher autonomy seek more maternal health care? Evidence from Eritrea and Ethiopia. Health Care Women Int 31(7): 599-620. doi:10.1080/07399331003599555. PubMed: 20526926. 20526926

[24] Bowers D (2008) Medical statistics from scratch: an introduction for health professionals. John Wiley & Sons.

[25] Central Statistical Agency (2007) Ethiopia Population and Housing Census Report. Central Statistical Agency, Addis Ababa, Ethiopia.

[26] Assefa N, Berhane Y, Worku A (2013) Pregnancy rates and pregnancy loss in Eastern Ethiopia. Acta Obstet Gynecol Scand 92(6): 642-647. doi:10.1111/aogs.12097. PubMed: 23384203. 23384203

